# Effect of experimental resin cements containing thio-urethane oligomers on the durability of ceramic-composite bonded interfaces

**DOI:** 10.1080/26415275.2019.1693274

**Published:** 2019-11-27

**Authors:** Lourenço Correr-Sobrinho, Ana Rosa Costa, Ana Paula P. Fugolin, Daniel Sundfeld Neto, Jack L. Ferracane, Carmem S. Pfeifer

**Affiliations:** a Department of Dental Materials, Piracicaba School of Dentistry, State University of Campinas, Campinas, Brazil;; b School of Dentistry, Biomaterials and Biomechanics, Oregon Health and Science University, Portland, Oregon;; c School of Dentistry, Department of Restorative Dentistry and Prosthodontics, Ingá University Center, Prostothontics, Brazil

**Keywords:** Resin cement, Thio-urethane oligomers, ceramic, thermocycling, load-cycling, bond strength

## Abstract

Thio-urethane oligomers improve conversion and mechanical properties of resin cements. The objective of this study was to evaluate the effect of resin cements formulated with thio-urethane (TU) oligomers on microtensile bond strength (µTBS) of ceramics to composites subjected to thermal/mechanical cycling.

**Methods:** BisGMA/UDMA/TEGDMA (50/30/20 wt%) containg 0 (control, EC) or 20 wt% aliphatic or aromatic thiourethane (HDDI and BDI, respectively) were mixed with CQ/amine (0.2/0.8 wt%) and 25 wt% 0.7um Ba glass. Rely X Ultimate (RU-3M ESPE) was used as the commercial control. The cements were sandwiched between ceramic (IPS e.max Press) and resin composite blocks (Filtek Supreme, 3 M-ESPE). Eight bonded blocks were produced per experimental group. Prior to bonding, ceramic surfaces were etched (20 s – 10% HF) and silanized. Composite surfaces were treated with Single Bond Universal (3 M ESPE). Specimens were stored for 24 h in distilled water at 37 °C, and then either tested immediately, or subjected to thermal (10,000, 5 °C and 55 °C) or mechanical cycling (300,000 cycles). Sticks (1 mm^2^, average of 25 sticks per block) were cut and tested for µTBS (1.0 mm/min). Data were analyzed with two-way ANOVA/Tukey’s test (*α* = 5%). Fracture surfaces were analyzed to determine failure modes.

**Results:** The µTBS for HDDI and RU was significantly higher than BDI and EC cements. BDI led to significantly higher µTBS than EC after 24 h, Tc and Mf. µTBS decreased significantly after thermal/mechanical cycling for all groups. Failure modes were predominantly adhesive or mixed.

**Significance:** The use of selected thio-urethane oligomers was able to increase the µTBS of composite-cement-ceramic specimens. Tc and Mf reduced µTBS for all resins cements.

## Introduction

All-ceramic systems have been widely used in dentistry to indirectly restore fractured/lost/decayed teeth, mainly due the growing demand for highly esthetic treatments. The long-term clinical success of the bond between dental ceramics and resin cements primarily depends on the composition of the ceramic material and the cementation procedure [1,2]. The luting procedure between the ceramic and the tooth is based on clinical adhesive strategies usually employing a resin cement [3,4]. The current gold standard for cementation of ceramics is the pretreatment of the ceramic with hydrofluoric acid (HF) etching, followed by application of a silane coupling agent and subsequently a resin cement [1,5–7]. The HF acid etches ceramic surfaces containing at least some glassy component, leading to increased surface area for micromechanical interlocking to enhance bond strength [5,6,8–10].

Conventional resin cements are extensively used in restorative dentistry because of their ability to chemically bond to the ceramic material and mechanically interlock the tooth substrate *via* hybrid layer formation. Due to the tapered nature of crown preparations for ceramic restorations and the constant physical and mechanical challenges to which these restorations are subjected, resin cements need strong adhesion to the tooth and the ceramic, adequate mechanical properties, solubility resistance to avoid cement degradation, and the ability to maintain the integrity of the interface [11,12]. It has been speculated that significant polymerization stress develops at the interface due to the volumetric shrinkage inherent in the polymerization process, which ranges from 1.7% to 5.3% [13], the high constraint within the thin cement layer (i.e. high C-factor) [14], and the fact that the methacrylate monomers in the cements undergo vitrification at early stages in the conversion reaction [15]. This stress imposed at the bonding interface may result in debonding between the resin cement and restorative material, resulting in gap formation that may compromise the durability of the restoration [16]. Therefore, producing dental cements with reduced polymerization stress and lower shrinkage, increased mechanical properties, and increased degree of conversion is highly desirable and the focus of recent studies [15,17,18].

The thiol-ene materials provide an attractive alternative to methacrylate resin systems for use in resin composites due to their potential for stress reduction *via* delayed gelation and reduced oxygen inhibition [19,20].Thiol-enes have been combined with other methacrylates in a ternary thiol-ene-methacrylate system to comply with the mechanical requirements for dental restorative applications. However, there is a potential for reduced mechanical properties due to the highly flexible nature of the thiol-carbon bond [20]. Because of that, thiol-isocyanate oligomers have been proposed as an alternative to conventional thiol-ene systems. When used in methacrylate networks in oligomeric form and with the presence of thio-carbamate bonds, more homogeneous networks have resulted, with increased toughness and fracture toughness [17,21,22] and degree of conversion [23], as compared with simple urethane-based systems. In addition, when experimental and commercial resin cements with thio-urethanes were used, improved bond strength of indirect composite and glass ceramic to dentin has been reported [17], as well as lower volumetric shrinkage and stress, and with an absence of odor concerns [18].

Clinically, cemented ceramic restorations are exposed to the oral environment where factors such as mechanical and thermal fatigue may influence their physical and mechanical properties [2]. Mechanical and thermal fatigue tests *in vitro* [2,4,24–26] have been used to simulate in an accelerated way the degradation mechanisms that may lead to a reduction in strength and increased risk of failure during prolonged exposure of materials in the oral environment. Mechanical fatigue simulates the chewing and clenching stress applications that cause the propagation of microscopic cracks within the material that may weaken it and lead to catastrophic failures [27–28]. Thermal cycling provides an alternative method to apply stresses at the bonded interface between different materials [29–30], potentially leading to degradation. Both tests have been used to simulate the occurrence of clinical failures of ceramic restorations [31].

Considering the high stress situation in thin cementation lines, and the potential for crack generation and propagation in the cement material, a rational step in improving the longevity of bonded prosthetic work is to modify the materials to overcome these issues. Thio-urethanes have been shown to both decrease the stress and improve fracture toughness in cement materials [15], but the effect of such improvements on the actual longevity of the bonded interface remains underexplored. In this study, mechanical and thermal fatigue were applied to bonded interfaces to simulate the clinically-relevant accelerated aging they would incur in the oral environment. Resin composites were used as the bonded substrate due to its modulus similar to dentin, with the advantage of eliminating the potential variation in a natural substrate. In summary, the aim of this study was to evaluate the effect of thio-urethane additives to resin cements on the µTBS of the ceramic IPS e.max Press and a dental composite (Z250), after imposing stress by mechanical fatigue (Mf) and thermal cycling (Tc). Two hypotheses were tested: (1) the use of thio-urethanes would improve µTBS; and, (2) the mechanical fatigue and thermal cycling would negatively affect the µTBS, but less so for the experimental cement containing the thio-urethane additives.

## Materials and methods

### Experimental resin cement

Two experimental thio-urethane oligomers were synthesized with formulations based on our previous work [18]. The aliphatic version was made by combining 1,6-hexanediol-diissocyante (HDDI) (aliphatic) with pentaerythritol tetra-3-mercaptopropionate (PETMP), while the aromatic version was made by combining 1,3-bis (1-isocyanato-1-methylethyl) benzene (BDI) (aromatic) with trimethylol-tris-3-mercaptopropionate (TMP). Both oligomers were produced with a 1:2 isocyanate:thiol molar ratio, resulting in pendant thiols. The reaction was carried out in solution (methylene chloride) at room temperature using triethylamine as a base in catalytic amounts. The oligomers were purified by precipitation in hexanes, the solvent was removed under vacuum, and the final products were characterized by 1 H-NMR and mid-IR spectroscopy [17]. The disappearance of the isocyanate peak at 2270 cm^−1^ and the appearance of resonance signals at 3.70 ppm were used as evidence of completion of the isocyanate reaction and thio-urethane bond formation, respectively [32].

The experimental dual-cured resin cement was composed of Bis-phenol A diglycidyl dimethacrylate (Bis-GMA), urethane dimethacrylate (UDMA) and tri-ethylene glycol dimethacrylate (TEGDMA; Esstech) in a 50:30:20 mass ratio (BisGMA-UDMA-TEGDMA materials). This monomer system was chosen based on a previous study [33] which showed enhanced bonding to ceramics compared to other resins. All monomers were purchased from Esstech (Essington, PA, USA). Photoinitiators were added to the resin matrix as follows: 0.2 wt.% of dl-camphoroquinone (Polysciences Inc., Warrington, PA, USA), 0.6 wt.% of a tertiary amine (EDMAB – ethyl 4-dimethylaminobenzoate; Avocado, Heysham, England), and 0.5 wt.% inhibitor (BHT – 2,6-di-tert-butyl-4-methylphenol; Sigma–Aldrich, St. Louis, MO, USA). Thio-urethane oligomers were added to the methacrylate organic matrix in proportions of 0 or 20 wt%. This proportion has been demonstrated to provide increased mechanical properties without affecting the material’s viscosity [33]. The cement without the thio-urethane served as an experimental control (EC).

A total of 25 wt% filler particles, consisting of a mixture of different fillers (85% silane-treated barium borosilicate glass filler with average size of 0.7 µm, Esstech; 15% silane-treated silica of 40 nm size, Aerosil OX50 from Degussa), was added to the monomer matrix with the aid of a centrifugal mixer (DAC 150 Speed mixer, Flacktek, Landrum, SC, USA) operated for 1 min at 2,500 rpm. This filler concentration was determined in a pilot study so that the film thickness of the experimental materials would be similar to that of the commercial control. All the procedures were performed under yellow lights.

A dual-cured resin cement (RU, RelyX Ultimate, 3 M ESPE, St. Paul, USA) composed of BisGMA/TEGDMA resin with filler content of 66 wt% (based on manufacturer data) was used in this study as a commercial control.

### Ceramic and composite resin blocks

Ninety-six ceramic blocks (8 mm × 8 mm × 3 mm thick) were made with IPS e.max Press (Ivoclar Vivadent, Schaan, Liechtenstein, shade LT D3) in accordance with the manufacturer`s instructions. Acrylic resin (Duralay, Reliance Dental MFG Company, Illinois, USA) patterns (8.5 mm × 8.5 mm × 3.5 mm) were invested in a phosphate based material (IPS PressVest Speed; Ivoclar Vivadent) and the acrylic resin was eliminated in an automated furnace (Vulcan A-550; Degussa-Ney, Yucaipa, CA, USA) by heating at 850 °C for 1 h. The ceramic ingots were then pressed into the molds at 915 °C in a furnace press (EP 600; Ivoclar Vivadent). After divestment, the ceramic square blocks were wet-polished with 600-, 1,200- and 2,000-grit silicon carbide abrasive papers (Buehler, Lake Buff, IL, USA) to obtain flat surfaces and further ultrasonically cleaned in deionized water for 15 min.

Ninety-six square composite blocks (8 mm long × 8 mm wide × 3 mm thick) were made with a commercial composite (Z250, 3 M ESPE, St. Paul, MN, USA, shade A3) and used as the bonding substrate. The composite block was used instead of natural teeth to avoid the intrinsic variability in the natural substrate, since the main purpose of this study was to evaluate potential differences in results derived from the cement formulation. One increment of composite 2 mm thick and a second of 1 mm thickness were inserted into a square elastomeric mold (Express STD; 3 M ESPE, St. Paul, MN, USA) to fabricate a 3 mm tall block. A Mylar strip was placed over the mold and manually pressed using a glass slide to remove excess resin composite. Each increment was photo-activated with the light guide positioned directly on the mylar for 20 s from the top surface using a LED source (Radii Plus; SDI Limited, Bayswater, Victoria, Australia) with an irradiance of 1,100 mW/cm^2^ as measured using a curing radiometer (Model 100, Demetron Research Corporation, Danbury, CT).

### Surface treatments and cementing the ceramic to the composite

The ceramic blocks were randomly divided into 12 groups (8 blocks per group): Groups 1 to 3 were bonded with experimental cement without thio-urethane (EC, Control); Groups 4–6 with experimental cement with 20 wt% of HDDI (Aliphatic); Groups 7–9 with experimental cement with 20 wt% of BDI (Aromatic); and, Groups 10–12 with commercial cement RelyX Ultimate (RU, 3 M ESPE). The ceramic surface was etched with 10% hydrofluoric acid (Dentsply, Petropolis, RJ, Brazil) for 20 s at room temperature, followed by rinsing with oil-free air-water spray for 30 s. One layer of a silane coupling agent (RelyX Ceramic Primer, 3 M ESPE, St Paul, MN, USA) was applied to the etched ceramic surface and left in contact for 60 s, followed by compressed air for 30 s. The composite surface was cleaned for 30 s with 35% phosphoric acid gel (3 M ESPE), and then rinsed with oil-free air-water spray for 30 s. One coat of Single Bond 2 (3 M ESPE) was applied on the composite surface and light-cured for 10 s (Radii Plus; SDI Limited). The ceramic blocks were then bonded to the composite blocks with the different cements and placed under 500 grams static load for 2 min. The excess cement was removed with a microbrush before light-curing for 40 s on each side of the specimen (four activations, Radii Plus), with the light guide tip positioned directly at the interface, simulating the clinical situation of a cementation. One final 40 s light exposure was delivered trhough the exposed composite surface.

### Thermal cycling (Tc) and mechanical fatigue (Mf) testing

All specimens were stored in distilled water at 37 °C for 24 h, and then Groups 1, 4, 7 and 10 were tested. The specimens in groups 2, 5, 8 and 11 were then subjected to Tc (10,000 cycles) in a thermal cycler (MSCT 3 – Marnucci ME, São Carlos, SP, Brazil) with water between 5 °C and 55 °C (dwell time of 30 s) and transfer time of 6 s between baths. In total, the specimens in these groups were kept immersed for 224 h (24 h or initial storage plus 200 h, which is the time it took for the entire Tc to be completed). The specimens of groups 3, 6, 9 and 12 were placed into a stainless steel box, and stabilized with a 1 mm layer of polyether impression material (Impregum F, 3 M ESPE, Seefeld, Germany) around the sides. These specimens were then subjected to Mf (ER37000 – ERIOS, São Paulo, SP, Brazil) of 300,000 sinusoidal cycles between 0 and 80 N in compression at 2 Hz in water. The 80 N load has been shown to be the average biting force in the posterior region for individuals with no para-functional habits [34,35]. The loading was accomplished with a stainless steel sphere of 8 mm diameter applied directly onto the central area of the ceramic, simulating a cusp tip. In total, the specimens in these groups were kept immersed for 66 h (24 h of initial storage plus 42 h, which is the time it took for the entire Mf to be completed).

### ***µ***TBS testing procedures

After storage, Tc and Mf testing, the ceramic/resin cement/composite blocks were cut perpendicular to the bonded interface to obtain sticks with 1 × 1 mm cross-sections using a water-cooled diamond blade (EXTEC Corporation, Enfield, CT, USA) in a low speed saw (Isomet 1000, Buehler, Lake Bluff, IL, USA). The cross-sectional area of the bond interface of each stick was measured using a digital caliper (Mitutoyo Corporation, Tokyo, Japan). Each stick was fixed to the grips of the testing jig using cyanoacrylate adhesive (Zapit, Dental Ventures of America, Corona, CA, USA) and the µTBS testing was conducted in a universal testing machine (EZ Test, EZS, Shimadzu, Tokyo, Japan) at 1.0 mm/min until failure. The fractured specimens were observed by optical microscopy at 40 × magnification (Olympus Corp, Tokyo, Japan), and the failure was classified into 5 modes: adhesive (mode 1); cohesive within ceramic (mode 2); cohesive within resin cement (mode 3); cohesive within resin composite (mode 4); and mixed, involving resin cement, resin composite and ceramic (mode 5). The results of failure mode classification were analyzed with the Chi-square test (*α* = 5%).

The µTBS (MPa) of each specimen was calculated by dividing the failure load by the cross-sectional area. The experimental unit was the ceramic/composite resin block, with each group containing eight blocks. After sectioning to produce the sticks, the outermost, oddly shapped sticks were discarded, and as a result, each block generated approximately eighteen sticks. No pre-test failures were observed, so the average was calculated for all the sticks of that block to represent the mean. Thus, the mean of the µTBS values in each group represented the mean of the eight experimental units. Data were tested for normality (Anderson-Darling) and equal variances (Levene) prior to being analyzed with two-way ANOVA (cements × aging condition). Multiple comparisons were performed using the Tukey post-hoc test (*α* = 5%).

## Results

Significant differences in µTBS for resin cements (*p* < .0001) and aging condition (*p* < .0001) were detected (Table 1). The interaction between the resin cements and aging condition (*p* = .6073) was not significant. Therefore, multiple comparisons were performed with separate Tukey’s tests within each material (as a function of aging condition) and within each aging condition (as a function of the material). For the results obtained after 24 h storage in distilled water, the mean values of µTBS of RelyX Ultimate, the experimental resin cement modified by HDDI and experimental resin cement modified with BDI were significantly higher than the experimental resin cement not containing thiourethane (EC) (*p* < .05). No statistical difference was found among BDI, HDDI and RelyX Ultimate (*p* > .05). For Tc and Mf, the mean values of µTBS of HDDI was significantly higher than resin cement modified by BDI and EC (*p* < .05). The RelyX Ultimate and resin cement modified by BDI presented significantly higher µTBS than EC (*p* < .05). No statistical difference was found between HDDI and RelyX Ultimate, and between BDI and RelyX Ultimate (*p* > .05). The mean values of µTBS obtained at 24 h for all resin cements were significantly higher than following Tc and Mf (*p* < .05). No statistical difference was found between Tc and Mf (*p* > .05). In comparison with the materials tested at 24 h, Tc and Mf led to a reduction of µTBS of 18% and 21.6%, respectively, for the BDI-modified material, 11.4% and 14.3%, respectively, for the HDDI-modified material, 17.6% and 21.2%, respectively, for the experimental control, and 14.7% and 21.4%, respectively, for RelyX Ultimate.

**Table 1.** Means of microtensile bond strength (µTBS) ± Standard Deviation (MPa) for the experimental control (EC), for the cements modified with aromatic (BDI) and aliphatic (HDDI) thiourethanes, and for the commercial control (RelyX Ultimate).

Resin Cements	µTBS (MPa)		
	24 h	Thermal fatigue	Mechanical fatigue
Experimental Control (EC)	30.2 ± 2.2 Ab	24.9 ± 2.5 Bc	23.8 ± 1.5 Bc
BDI	36.5 ± 1.1 Aa	29.9 ± 1.7 Bb	28.6 ± 1.7 Bb
HDDI	38.6 ± 1.9 Aa	34.2 ± 3.3 Ba	33.1 ± 1.7 Ba
RelyX Ultimate	38.8 ± 2.5 Aa	33.1 ± 2.1 Bab	30.5 ± 2.6 Bab

Values followed by the same lower-case superscript within the same column and upper-case superscript in the same row are statistically similar (*α* = 5%).

Compared to the un-modified experimental control (EC), the addition of the aliphatic version of the thiourethane led to increases of 27.8%, 37.4% and 39.1% in µTBS after 24 h storage, following Tc and Mf, respectively, and the addition of the aromatic version led to an increase of 20.8%, 20.1% and 20.1% in µTBS, for the same conditions.

Failure mode results are shown in Table 2. Though there was a trend for increased adhesive (mode 1) failures after Tc and Mf as compared to 24 h, the Chi-square test of the failures modes within each conditions showed no significant association between the modes of failures and resin cements in the three aging conditions (*p* > .05). A predominance of failures were adhesive (mode 1) or mixed (mode 5).

**Table 2.** Failure Mode Analysis of the Debonded Specimens - % among groups and (number of sticks)^a^.

Resin	Aging condition	Failure Modes				
Cement		Mode 1	Mode 2	Mode 3	Mode 4	Mode 5
24 hours	EC	39 (56)	4 (6)	13 (19)	4 (6)	40 (57)
	BDI	37 (50)	6 (8)	8 (11)	8 (11)	41 (56)
	HDDI	32 (46)	8 (12)	14 (20)	8 (12)	38 (54)
	Rely X Ultimate	33 (47)	5 (7)	15 (22)	8 (12)	39 (56)
		*p* = .6337				
Tc	EC	48 (69)	4 (6)	10 (14)	5 (7)	33 (48)
	BDI	47 (68)	7 (10)	18 (26)	2 (3)	26 (37)
	HDDI	45 (61)	5 (7)	16 (22)	7 (9)	27 (37)
	Rely X Ultimate	42 (60)	9 (13)	12 (17)	6 (9)	31 (45)
		*p* = .3072				
Mf	EC	51 (73)	3 (4)	18 (26)	4 (6)	24 (35)
	BDI	49 (71)	7 (10)	16 (23)	2 (3)	26 (37)
	HDDI	49 (71)	4 (6)	17 (24)	4 (6)	26 (37)
	Rely X Ultimate	45 (61)	4 (6)	11 (15)	2 (3)	38 (51)
		*p* = .3552				

a Failure modes: adhesive between cement and ceramic (mode 1); cohesive within ceramic (mode 2); cohesive within resin cement (mode 3); cohesive within composite resin (mode 4); and mixed, involving resin cement, composite resin and ceramic (mode 5). No adhesive failures were observed between the cement and the composite.

## Discussion

The quality and durability of the bond between the resin cement and the ceramic material is essential for the clinical success of all-ceramic restorations [2]. Many strategies have been proposed and clinically tried to maintain that bond, including specific treatments to promote micromechanical or chemical retention of the ceramic restorations to the tooth [36]. Surface modifications have been employed to improve the bond strength between resin cements and ceramic restorations [37]. These modifications include some type of acid etching to increase surface area and create micromechanical retention, followed by the application of coupling agents with the intent to create covalent bonds between the ceramic material and the resin cement layer. The polymerized cement helps seal the margin of the restoration and provides additional retention for the crown [38].

The first hypothesis of this study, which stated that the use of thio-urethanes as additives in resin cements would improve µTBS, was confirmed. The results showed that the microtensile bond strength of the formulation of methacrylate-base resin cement containing the aliphatic and aromatic versions of the thio-urethane oligomers were significantly higher than the values observed for the experimental control. This was true after 24 h storage, as well as after each aging condition (mechanical and thermal fatigue). The use of thio-urethane-modified materials has previously been shown to improve the properties of resin cements, with increased mechanical properties and degree of conversion, and reduced polymerization stress [15,18]. These previous studies have correlated those findings to increased degree of conversion, as well as delayed gelation and vitrification and the formation of more homogenous polymer networks through the addition of these oligomers, all of which lead to improved properties, especially fracture toughness [21]. This may help explain the increase in bond strengths observed in this study.

Though this effect was observed for both oligomers in comparison to the control, the aliphatic version presented bond strength values that were significantly higher than those of the aromatic version after aging aging condition (mechanical and thermal fatigue). One explanation for the more significant increase in bond strength for the aliphatic version is the potentially greater flexibility of the aliphatic oligomeric chains, which would increase the toughness of the cement layer, as demonstrated in a previous study in which higher fracture toughness and degree of conversion was achieved for a cement containing this specific version of the oligomer [18]. The great flexibility of the aliphatic oligomer version is due to the thio-urethane bonds, but also from the absence of aromatic rings or other rigid molecular substitutions. The increased flexibility can not only increase toughness, but can also facilitate chain-transfer reactions within the methacrylate matrix during the polymerization, leading to delayed gelation/vitrification, more homogeneous network formation and potentially lower residual bulk stress [39]. Indeed, for each aging condition, the aliphatic version led to bond strengths nearly one-third greater than in the experimental control. For the aromatic version, the increase in bond strength compared to the control was only about one-fifth. However, for 24 h storage, no statistical difference was found between aliphatic and aromatic versions. This result is in agreement with those of a previous study, which found no statistical difference in the mechanical properties between the aliphatic and aromatic versions of the oligomers in experimental resin composites [40], demonstrating that these effects are dependent on the relative composition of the material to which the oligomers are added and the type of aging. Therefore, generalizations need to be made with care.

Clinically, the flexibility of the structure of the aliphatic oligomer version can be important for resin cements. According to a previous study, a thin layer of cement placed over a tapered preparation constantly subjected to oblique loads, tension, compression and fatigue has shown the formation of micro-defects within its structure [18]. The durability of the bond between a resin luting agent and a silanated ceramic surface can be influenced by the presence of water and organic solvents, thermal change, cyclic mechanical loading, and pH variations in the oral environment [2,37]. All of these factors are able to induce physico-chemical alterations in dental materials [41]. In fact, previous studies showed that ceramics restorations demonstrated a susceptibility to stress corrosion when submitted to thermal and mechanical fatigue under wet conditions, leading to decreased resistance to catastrophic fractures [31,42,43]. Therefore, this study sought to test the thio-urethane modified materials under conditions that at least partially simulate stresses in the oral environment, such as thermal and mechanical fatigue. Indeed, the two aging conditions were shown to significantly decrease the bond strength of all of the resin cement groups as compared to the simple storage in water for 24 h. Thus, the second hypothesis that stated that the mechanical and thermal fatigue would affect the bond strength was also confirmed.

It is important to note a few things about the experimental design. During Tc and Mf, the specimens were also immersed in water, and therefore, exposed to hydrolytic conditions for longer than the specimens stored for only 24 h. This longer storage alone might have contributed to further degradation of the interface, and its effects cannot be de-coupled from the actual thermal and mechanical fatigue imposed on the specimens. In addition, the bonded blocks were cycled as whole units, and only after the different storage periods were sticks obtained for microtensile bond strength testing. The values presented in the tables represent averages of all of those sticks, including the ones closer to the edges (and therefore, more exposed to water and variations of temperature during Tc) and closer to the center (and therefore, closer to the point of load application during Mf). It is acknowledged that in the clinical situation the stresses (shear and tensile) are concentrated in the area of the margin, which is also more prone to contact with saliva and the oral environment, making that the most fragile region of the restoration. It is also acknowledged that the load distribution in the mouth is far more complex than the centered load applied here. This is a potential limitation of the study. Therefore, we opted for not analyzing the sticks according to their region of origin in the restoration, but rather considered the average value obtained by the block. It is important to note that the variance in the bond strengths for the 24 h, Tc and Mf specimens did not differ, supporting the approach.

When compared to the 24 h storage group, the bond strength for the experimental control decreased by roughly 18% and 21% after thermal and mechanical fatigue, respectively. The aromatic oligomer led to similar levels of bond strength reduction after aging. The aliphatic oligomer, however, led to somewhat lesser reductions of 11 and 14% after the thermal and mechanical fatigue respectively. Interestingly, the commercial material, showed reduction in bond strength values of 15 and 21%, after thermal and mechanical fatigue, respectively, which was comparable to the experimental control. This indicates that the aliphatic version of the thio-urethane may be a viable additive to improve the bond strength durability and especially after further optimization experirments are performed. The reduced bond strength observed in this study after mechanical fatigue is in agreement with others showing that when the specimens were subjected to a cyclical loading under wet conditions, the bond strength significantly decreased as a function of the the propagation of small cracks at the interface between the resin cement and ceramic surface [2,31]. In addition, thermal stresses introduced by variations in temperature can be exacerbated by the combination of materials with different thermal conductivities and thermal expansion coefficients when bonded together [44]. Also, different elastic moduli of the materials at the bonded interface can cause stress concentrations which may contribute to its degradation [25]. All of these factors, in addition to the degradation effects brought on by the continued uptake of water during the tests, contributed to the reduction in bond strengths observed.

While the interface-related factors discussed so far certainly played a major role in the results, the potential degradation of the bulk mechanical properties of the resin cements when subjected to thermal and mechanical loading cannot be neglected as a factor affecting the bond strength. The reduction of mechanical properties of the resin cement is a result of a continuous water sorption [2]. Previous studies have shown that the sensitivity of a resin composite depends on the filler type and concentration, volume fraction of intrinsic nanometer-sized pores, degree of monomer conversion and overall monomer chemistry [45,46]. Studies have demonstrated that increasing the ratio of tri-ethylene glycol dimethacrylate (TEGDMA) and urethane dimethacrylate (UDMA) in relation to bisphenol-A-glycidyl methacrylate leads to increased water sorption [47,48]. It is likely that the presence of TEGDMA and UDMA in the experimental resin and TEGDMA in the resin cement RelyX Ultimate may have contributed to the acceleration of water sorption and significantly decreased the bond strength for all resin cements after prolonged exposure to thermal and mechanical stresses.

The analysis of failure modes showed a predominance of mixed failures (mode 5) for all groups subjected to bond strength testing after 24 h (Table 2). Conversely, adhesive failures (mode 1) were more frequent after thermal and mechanical fatigue, indicating poorer bond quality. Interestingly, this seems to indicate that the bulk integrity of the cements in general was better preserved than the actual bond to the ceramic. It is also interesting that the number of cohesive failures in the ceramic were relatively high, which may have been due to microcrack generation during specimen preparation, especially the cutting procedure, which would likely be exacerbated by stress concentration during the tensile test.

## Conclusion

The present study demonstrated that the use of thio-urethane oligomers as additives in the formulation of experimental resin cements led to increased bond strength to ceramic materials. This effect was dependent on the oligomer type, with aliphatic oligomers being more effective. Thermal and mechanical loading reduced the bond strength, as expected. It is important to highlight that, since the additon of thio-urethane oligomers to resin cements requires no change in traditional clinical procedures, the translation of this technology to commercial products could be relatively simple.
